# Factors associated with bariatric surgery rates in federative units in Brazil

**DOI:** 10.11606/s1518-8787.2022056004133

**Published:** 2022-11-18

**Authors:** Ivan Augusto Cecilio e Silva, Cassia Kely Favoretto, Leticia Xander Russo

**Affiliations:** I Universidade Federal do Rio Grande do Sul Faculdade de Ciências Econômicas Programa de Pós-Graduação em Economia Porto Alegre RS Brasil Universidade Federal do Rio Grande do Sul. Faculdade de Ciências Econômicas. Programa de Pós-Graduação em Economia. Porto Alegre, RS, Brasil; II Universidade Estadual de Maringá Centro de Ciências Sociais Aplicadas Departamento de Economia Maringá PR Brasil Universidade Estadual de Maringá. Centro de Ciências Sociais Aplicadas. Departamento de Economia. Maringá, Paraná, PR, Brasil; III Universidade Federal da Grande Dourados Faculdade de Administração, Ciências Contábeis e Economia Departamento de Economia Dourados MS Brasil Universidade Federal da Grande Dourados. Faculdade de Administração, Ciências Contábeis e Economia. Departamento de Economia. Dourados, MS, Brasil

**Keywords:** Bariatric Surgery, Healthcare Disparities, Sociodemographic Factors, Socioeconomic Factors

## Abstract

**OBJECTIVE:**

To analyze the socioeconomic, demographic and health management factors associated with bariatric surgery rates performed by the Brazilian Unified Health System (SUS) in the federative units in Brazil.

**METHODS:**

Description and analysis of bariatric surgeries rates (per 100,000 inhabitants) performed by SUS in adults from 18 to 65 years old, in the 27 federative units of Brazil, between 2008 and 2018; thus, the econometric methodology of count panel with negative binomial distribution (population-averaged, fixed effects and random effects) was used. Socioeconomic and demographic factors were also investigated, considering the real gross domestic product *per capita*, the average years of study of adults and life expectancy at birth, and those of health management, given the primary health care coverage, the rate of digestive system surgeons and the rate of hospitals accredited in high complexity care to patients with obesity in the SUS.

**RESULTS:**

In regional terms, the performance of public bariatric surgeries in Brazil over the period analyzed suffered a great disparity; the procedures happen mostly in the South and Southeast regions, and scarcely in the North region. Moreover, we found a positive relationship between the rate of bariatric surgeries and life expectancy, the rate of digestive system surgeons and the rate of hospitals accredited in high complexity care; however, the average number of years of adult study and coverage of primary health care is a negative association regarding real gross domestic product *per capita*.

**CONCLUSION:**

In the period analyzed, the investigated factors explained the rate of bariatric surgeries. Therefore, to train specialized health professionals, the accreditation of hospitals according to the legal framework, preventive actions of primary care, and socioeconomic and demographic factors, conditioning for the offer of surgical treatment by the SUS were crucial. Thus, these are all relevant factors for the formulation of public policies in this area.

## INTRODUCTION

Bariatric surgery is an effective and good cost-benefit intervention for the treatment of morbid obesity in adults (BMI ≥ 40 kg/m^2^ — Grade III), especially for patients with comorbidities such as diabetes and hypertension^1^. Brazil, for example, is the second country performing the most bariatric surgeries in the world, accounting about 17% of the procedures among all countries, behind only the United States^2^. However, private health cares do only about 90% of these procedures, while 75% of the population depends exclusively on the public health system^3^. The demand for surgical treatment increases, since morbid obesity expands in the Brazilian population — more frequent among women (1.3% in 2006 and 1.9% in 2017) than among men (0.9% in 2006 and 1.4% in 2017) — reaching 1.1% in total (men and women) in 2006, and 1.7% in 2017^4^.

Regarding public policies, the Brazilian Unified Health System (SUS) included bariatric surgery in the table of procedures in 1999, and in the following year the accreditation of hospitals began to perform it^5^. In 2007, the Ministry of Health authorized three procedures that reduce more than 60% of initial excess weight from patients^6^: vertical banded gastroplasty, Roux-Y gastric bypass, and biliopancreatic shunting, or duodenal switch. In 2013, the SUS started to do vertical gastrectomy in sleeve^7^, and, from 2017, incorporated bariatric surgery by laparoscopy^8^.

Between 2008 and 2019, the number of bariatric surgeries performed by the SUS, in adults from 18 to 65 years old, showed an increasing trend from 3,158 to 12,432, that is, an expansion of 294% in a little more than a decade. Based on this information, most regions of the country showed absolute positive variation: in the South, the total expanded from 1,372 to 8,191 surgeries (497%); in the Midwest, from 131 to 372 (184%); in the Southeast, from 1,230 to 3,240 (163%); and in the Northeast, from 375 to 587 (57%). Only in the North region the rate of surgeries was negative, decreasing from 50 to 42, 16% over the period analyzed^9^.

However, in the SUS, the supply of bariatric surgeries is still scarce; funding is limited and, therefore, many specialized public hospitals do not perform the minimum number of these surgeries, of 96 per year or, on average, eight per month, established by the Ministry of Health^10,11^. Moreover, the disparities in access to this type of procedure in Brazil limit the country to a small portion of patients who can benefit from treatment against morbid obesity, constituting an economic issue beyond a health issue.

Recent studies show that there are several factors associated with the behavior of bariatric surgery rates in the international context^12^. Some authors highlight socioeconomic and demographic constraints, such as gross domestic product (GDP) *per capita*, inflation and unemployment rate, legislative changes, poverty and education^3,12,13^. Other authors highlight the health aspects: prevalence of obesity, Body Mass Index (BMI), health expenses *per capita*, presence of bariatric surgeons and specialized hospitals^3,14^.

In Brazil, the current literature is based mainly on descriptive analyses of the evolution in the number and types of procedures, focusing on the profile of patients, that is, on their age, gender, ethnicity and comorbidities^10,11,17^. A recent study^3^ analyzed the correlation between the proportion of bariatric surgeries performed by the SUS and macroeconomic variables, however, the method employed — Spearman’s correlation coefficient — allowed only the evaluation of the intensity and direction of the association between two variables. Thus, the present study aims to contribute to the national and international literature by filling the gap exposed, seeking to analyze the socioeconomic, demographic and health management factors associated with the rates of bariatric surgeries performed by the SUS in the federative units of Brazil between 2008 and 2018.

## METHODS

This is a descriptive and analytical study that uses data on the number of bariatric surgeries performed by SUS, in adults from 18 to 65 years old, in the 27 federative units of Brazil, between 2008 and 2018. All data were collected from secondary sources and without identifying individuals, according to ethical research criteria. The Chart presents a description of the variables used, highlighting two groups of explanatory factors: socioeconomic and demographic (i), and health management (ii).

**Chart:** Description of the variables (dependent and explanatory) used in the research, federative units of Brazil, 2008–2018.

Variable	Description	Source
bariatricas	$\text{ Rate of bariatric surgeries performed by SUS (per }100,000\text{ inhabitants) = }\left[\left(\frac{\text{ number of bariatric surgeries }}{\text{ estimated population by FU }}\right)\times 100,000\right]$	SIH/ Datasus^9^
Socioeconomic, demographic and environmental factors		
pibpc	$\text{ Real GDP per capita -in}1,000\text{ reais (deflated by the IPCA at }2018\text{ prices) }=\left[\frac{{IPCA}_{2018}}{{IPCA}_{\text{year }}}\times \frac{\left(\text{ state }GDP÷1,000\right)}{\text{ estimated population by FU }}\right]$ .	Contas Regionais/ IBGE^18^
anos_estudo	$\text{Average years of study in adults (18 to 65 years) =}\left[\frac{\text{total of study years}}{\text{total of adults}}\right]$	PNAD, PNAD Contínua /IBGE^20-21^
expec_vida	Life expectancy at birth (in years)	TCM/IBGE^22^
Health management factors		
atencao_basica	Percentage of the population covered by family health strategy and traditional primary care teams, equivalent and parameterized regarding population estimation (decimal ratio).	e-Gestor AB^24^
cirurgioes	$\text{Rate of digestive system surgeons who serve the SUS (per 100,000 inhabitants) =}\left[\left(\frac{\text{ digestive system surgeons who serve the SUS }}{\text{ estimated population by FU }}\right)\times 100,000\right]$	CNES/ Datasus^25^
hospitais	$\text{Rate of hospitals accredited in high complexity care (SUS) to individuals with obesity (per 100,000 inhabitants) =}\left[\left(\frac{\text{ accredited hospitals by SUS that perform bariatric surgery }}{\text{ estimated population by FU }}\right)\times 100,000\right]$	SIH/ Datasus^9^

GDP: gross domestic product; IPCA: National Broad Consumer Price Index; SIH: ……; IBGE: ……………; PNAD: ……….TCM: ……….; AB: ……….; CNES: National Registry of Health Establishments.

Information regarding the number of bariatric surgeries was obtained via the Hospital Information System^9^ (SIH), available on Datasus. In the data collection, the TabWin-SUS software was used applying the following parameters: i) federative hospitalization units per year of processing and frequency; ii) period between January 2008 and December 2018; iii) age between 18 and 65 years old; iv) procedures performed from gastrectomy with or without duodenal bypass (0407010122), gastroplasty with intestinal bypass (0407010173), vertical banded gastroplasty (0407010181), vertical gastrectomy in sleeve (0407010360), laparoscopy bariatric surgery (0407010386), and diagnosis CID10 (category) referring to E66 (obesity). Bariatric surgery rates were obtained by dividing the number of procedures performed, per federative units and year, by its estimated resident population. This rate was measured per 100,000 inhabitants, according to previous studies in the area^11,12^.

The age indicated in the Brazilian legal framework for bariatric surgery is mostly between 18 and 65 years old^6^, with some exceptions not considered in this analysis: i) adolescents between 16 and 18, if the growth epiphyses are already consolidated, and ii) elderly over 65, considering carefully the risk-benefit relationship in each case^7^. On the other hand, the aforementioned procedures were also selected based on this milestone, considering that vertical sleeve gastrectomy was included in the SUS procedure table from 2013^7^, and laparoscopy in 2017^8^.

The real GDP *per capita* (in 1,000 reais) was used to capture the economic level of Brazilian federative units. This variable is important, because macroeconomic cycles and income level can influence the functioning of the SUS and the performance of bariatric surgeries^3^. This factor was obtained from the System of Regional Accounts^18^ of the Brazilian Institute of Geography and Statistics (IBGE) and deflated at 2018 prices (end of the period) based on the National Broad Consumer Price Index (IPCA)^19^.

The average number of study years of adults between 18 and 65 years old represented the social factor, in which educational inequality is shown in the federative units of Brazil. Because the educational level can influence the morbid obesity rates of the population and the demand for treatments such as bariatric surgeries^12^. The National Household Sample Survey (PNAD)^20^ was used as a source for the data of the years 2008, 2009, and from 2011 to 2015, the Annual Continuous PNAD^21^ for 2016, 2017 and 2018. For 2010, due to the unavailability of the data, the average between the immediately preceding and the following years was adopted as an estimate.

The demographic factor corresponded to life expectancy at birth, in years, which is based on the complete mortality tables, per year and federation unit, according to IBGE^22^. Life expectancy is fundamental to explain the proportion of adults with obesity and, therefore, bariatric surgeries, to the extent that health and well-being care also impacts the process of population aging^23^.

The coverage of primary care is a health management factor obtained by the Information and Management System of primary care^24^ (e-Gestor), representing the percentage of the population that is assisted by teams of the Family Health Strategy and traditional primary care, essential for prevention and early diagnosis of obesity in federative units.

The rate of digestive system surgeons who serve the SUS, measured per 100,000 inhabitants, is a way to analyze the ability of the public system to offer bariatric surgeries, conditioned to the availability of specialists in the procedure, following the literature^16^. These data were obtained from the National Registry of Health Establishments^25^ for human resources, which presented a great monthly variability at the rate mentioned above, and the calculation of its annual average is necessary.

The rate of hospitals accredited to high complexity care to individuals with obesity (per 100,000 inhabitants) represents the infrastructure — human resources and equipment — necessary to perform bariatric surgery procedures, according to Ordinance No. 425 of 2013^7^ and the international literature^12^. Data were collected from SIH^9^ using the TabWin-SUS software, by applying the frequency parameter according to the hospital.

Finally, the resident population rate per federative unit and year of analysis, according to IBGE estimates^26^, was used to calculate the indices of bariatric surgeries, digestive system surgeons who serve the SUS and hospitals accredited to high complexity care to individuals with obesity. This was accomplished when dividing these variables by the intensity indicator of each of the units, that is, by the number of inhabitants.

By hypothesis, it is expected that socioeconomic, demographic and health management factors are related to the rate of bariatric surgeries performed by the SUS between 2008 and 2018, and that they explain the regional differences observed in each federative units, considering the amount of these procedures.

Due to the characteristics of the studied phenomenon, such as the presence of heterogeneity among the units of the Brazilian federation over time (longitudinal data), the strong concentration of “zeros” — units that did not perform any surgery — and the overdispersion — variance greater than the average, we opted for the econometric methodology of count panel with negative binomial distribution (population-averaged, fixed effects and random effects)^27^. Thus, the aim of this study is to model the countable number of bariatric surgeries performed per 100,000 inhabitants, considering the limitation imposed by the scarcity of other methodologies for the zero-inflated count panel, more commonly applied only in cross-section form — for a single year. Finally, all the research data were organized in a spreadsheet and the estimates were made from the *Stata Software* 13.

## RESULTS

Table 1 shows the descriptive statistics of the variables — dependent and explanatory — for the federative units of Brazil, from 2008 to 2018. The average rate of bariatric surgeries performed by SUS was 2.44 per 100,000 inhabitants, with a maximum value of 58.46 per 100,000, registered in Paraná in 2018. The minimum value 0, no surgery, occurred in 85 observations during the period, in which ten were units of the federation, in 2008, and seven, in 2018. We noticed that the standard deviation is greater than the mean (6.328 > 2.439). Therefore, variance — standard deviation squared — also indicates overdispersion of the data. Thus, the relative dispersion was high — the coefficient of variation was 259.45%, indicating that this procedure between the analyzed areas has inequalities in performing.

**Table 1:** Descriptive statistics of the variables (dependent and explanatory) used in the research, federative units of Brazil, 2008–2018 (n = 297).

Dependent variable/factors	Mean	Standard deviation	Minimum value	Maximum value	CV (%)
Rate of bariatric surgeries performed by SUS (per 100,000 inhabitants)	2.439	6.328	0.000	58.455	259.45
Socioeconomic, demographic and environmental factors					
Real GDP *per capita* (in 1,000 reais)	28.082	15.304	9.160	89.780	54.50
Average adult education (in years)	9.158	0.936	6.900	11.700	10.22
Life expectation at birth (in years)	73.539	2.626	68.100	79.700	3.57
Health management factors					
Primary health care coverage (decimal ratio)	0.763	0.130	0.405	0.998	17.04
Rate of digestive system surgeons who serve the SUS (per 100,000 inhabitants)	0.776	0.622	0.067	2.679	80.15
Rate of hospitals accredited in the HCC for individuals with obesity in the SUS (per 100,000 inhabitants)	0.035	0.037	0.000	0.159	105.71

SUS: Brazilian Unified Health System; GDP: gross domestic product; CV: coefficient of variation; HCC: high complexity care.

The average real GDP *per capita* was R$ 28,082.00 between 2008 and 2018, with a minimum value of R$ 9,160.00 in Piauí in 2008, and a maximum of R$ 89,780.00 in the Federal District in 2010. The average years of study of adults in the period analyzed was approximately 9.2 years, with the lowest value of this factor (6.9 years) observed in Alagoas in 2008, and the highest (11.7 years) in the Federal District in 2018. The average life expectancy — demographic factor — corresponded to 73.5 years, reaching the minimum age of 68.1 years in Maranhão in 2008, and a maximum of 79.7 years in the state of Santa Catarina in 2018. Since the coefficient of variation of these three factors was 54.50%, 10.22% and 3.57%, respectively, the main disparity between the federative units was economic.

Regarding health management factors, between 2008 and 2018, the coverage of average primary health care was 76.3%, with a minimum of 40.6% in the Federal District in 2008, and a maximum of 99.8% in Piauí in 2018. The average rate of digestive system surgeons in the SUS was 0.77 per 100,000 inhabitants between 2008 and 2018, with a minimum value of 0.067 per 100,000 inhabitants in Rondônia, in 2008, and a maximum of 2.67 per 100,000 inhabitants in Paraná in 2018. The rate factor of hospitals accredited in high complexity care for people with obesity in the SUS recorded an average of 0.035 per 100,000 inhabitants, with the highest value of this variable of 0.159 per 100,000 inhabitants, identified in Paraná in 2018. Thus, the results of the coefficients of variation of the first (17.04%), second (80.15%) and third (105.71%) conditioning factors showed that the health sector presented differences in behavior between the units of the Brazilian federation.

**Figure 1:**
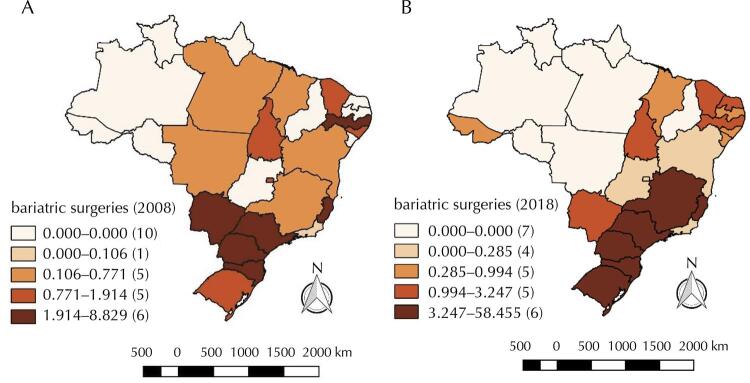
Quantile maps of the rate of bariatric surgeries (per 100,000 inhabitants), federative units of Brazil, 2008 (a) and 2018 (b).

In regional terms, we noticed an inequality in the rate of bariatric surgeries during the period. Figure 1 shows the distribution of rates on the map of Brazil for the years 2008 and 2018, showing the geographical evolution from the beginning to the end of the analysis. The concentration is higher in the South and Southeast regions, especially in 2018, while the North region has a lower supply of the procedure. The states of Acre, Rio Grande do Norte, Paraíba, Sergipe and Goiás did not perform bariatric surgery by SUS in 2008; however, in 2018, they had already implemented it. Pará and Mato Grosso performed this type of procedure in 2008 but stopped in the last year of analysis. Five other states, Rondônia, Amazonas, Roraima, Amapá and Piauí, did not register any public bariatric surgery during the years studied.

The variable “bariatric surgeries” refers to the rate of bariatric surgeries performed by the Brazilian Unified Health System (SUS) (per 100,000 inhabitants), “study_years” is the average years of study in adults (in years); “life_expec” is life expectancy (in years); “primary_care” is the population coverage of primary health care (on decimal grounds); “surgeons” is the rate of digestive system surgeons in the SUS (per 100,000 inhabitants); “hospitals” is the rate of hospitals accredited in the high complexity care for obese people in the SUS (per 100,000 inhabitants).

**Figure 2:**
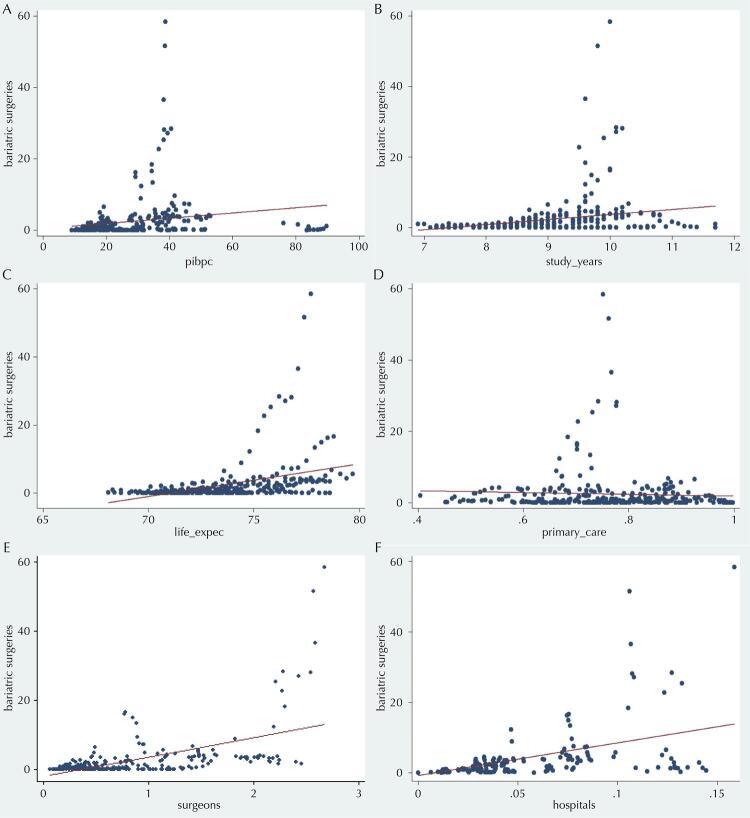
Dispersion diagrams between the dependent variable and the explanatory variables, federative units of Brazil, 2008–2018 (n = 297).

Figure 2 shows the dispersion diagrams between the dependent variable and each of the explanatory factors. The index of bariatric surgeries performed by the SUS presented a positive linear adjustment with socioeconomic, demographic and health management factors, except with the coverage of primary care.

Table 2 shows the results for count panel with Negative Binomial distribution and with robust standard error (population-averaged models, random effects and fixed effects). In the count panel by fixed effects, Rondônia, Amazonas, Roraima, Amapá and Piauí, did not enter the estimate, because only zeros composed the dependent variable in all 55 observations; therefore, the total number of observations was lower in this model (n = 242).

**Table 2:** Results of models estimates in negative binomial panel with robust standard error, federative units of Brazil, 2008–2018.

Variables	Population-averaged	Random effects	Fixed effects
Constant	-26,587^c^	-13,035	13,437
	(7,511)	(373,103)	(379,157)
GDP *per capita* (real)	-0,052^c^	-0,045^c^	-0,042^b^
	(0,012)	(0,011)	(0,021)
Years of study of adult (mean)	-0,180	-0,362^a^	-0,102
	(0,232)	(0,219)	(0,344)
Life expectancy	0,429^c^	0,487^c^	0,092
	(0,125)	(0,089)	(0,324)
Coverage of primary health care	-3,933^c^	-2,774^b^	-3,676
	(1,254)	(1,290)	(2,403)
Rate of digestive system surgeons who serve the SUS	0,674^c^	0,642^c^	0,671^b^
	(0,218)	(0,204)	(0,310)
Rate of hospitals accredited in the HCC for individuals with obesity in the SUS	16,252^c^	13,483^c^	10,518^c^
	(3,253)	(2,339)	(2,486)
Dummies per year	Yes	Yes	Yes
Standard error	Robust	Robust	Robust
n	297	297	242
Hausman test	-	0,978	

GDP: gross domestic adult; SUS: Brazilian Unified Health System; HCC: high complexity care.

Standard error in parentheses:

^a^ p < 0.1

^b^ p < 0.05

^c^ p < 0.01

Hausman’s test was insignificant (p > 0.05), thus, the random effects model was more appropriate than fixed effects for analysis of the results. In the first model, all factors were statistically significant at the level of 10%. GDP *per capita*, years of study and coverage of primary health care had negative associations with the rate of bariatric surgeries. However, life expectancy, the rate of digestive system surgeons and the rate of hospitals accredited in High Complexity Care had positive associations.

## DISCUSSION

From the results, we verified the negative effect of the economic factor — real GDP *per capita* — regarding the index of bariatric surgeries per federative unit. This means an increase in the economic level associated with the reduction in the supply of this procedure, possibly because strategic actions to prevent obesity tend to be adopted in the most economically developed areas, prejudicing low-income^28^ populations — nevertheless, another study^3^ did not find a relevant correlation between the proportion of public bariatric surgeries and the annual rate of GDP variation in Brazil between 2003 and 2017. However, this article differs from the previous approach in some important respects: in the use of the count panel; in the incorporation of several variables, including dummies for control of each year; and in the analysis at the level of the federative units, capturing the local particularities.

On the one hand, schooling — an average of years of study — was also negatively associated with the offer of bariatric surgeries by the SUS in each federative unit, contrary to what points out^12^ a study conducted for the USA, from 2002 to 2012, in which the educational factor was insignificant for the process of diffusion of bariatric surgeries in the American states. Lower levels of schooling can be a risk factor for obesity, especially for women^29^, who seek the most the procedures offered by the SUS, comprising 85% of the cases^17^.

On the other hand, given life expectancy, the demographic factor had a positive and significant impact on the index of bariatric surgeries in Brazilian federative units between 2008 and 2018. Since the population has aged, health and well-being care measures have become necessary, especially regarding severe morbid obesity and the supply of surgeries^23^. This effect implies that, if the aging process of the population in the federation units, over time, is unhealthy — a consequence of sedentary lifestyle and poor diet throughout life — morbid obesity tends to increase, expanding the demand for the medical procedure.

In these circumstances, the increase in coverage of primary health care teams contributed to the reduction of bariatric surgery rates in federative units, reinforcing the importance of this public policy strategy for the prevention and control of morbid obesity rates in the country. The effectiveness of the comprehensive and intra-sectoral approach to obesity in the SUS, within the scope of the lines of care for chronic diseases, reinforces the link between the coverage of primary health care and the demand for medium or high complexity services^30^.

The positive impact of the rate of digestive system surgeons on the index of bariatric surgeries performed by the Unified Health System in federative units indicated that this factor is of fundamental importance for the offer of the procedure in the country, especially in the regional context. Therefore, the hiring, valorization and training of these professionals in the public health system are essential to minimize the problem of morbid obesity^11^. These results conform to the 2013 U.S. case study, in which effective treatment for morbid obesity appeared limited by the number of trained surgeons between different areas of this country^16^.

Equally, the accreditation rate of hospitals in high complexity care for obese individuals showed a positive association with the rates of bariatric surgeries performed in the public health system of Brazil. Therefore, the expansion of specialized and accredited hospitals in the states is an essential factor to expand the supply of the procedure, particularly in the less assisted regions^10^. Although Rondônia, Amazonas, Roraima, Amapá and Piauí did not register public bariatric surgery during the period analyzed, the states recorded individuals with morbid obesity, despite the lack of specialized services accredited by the SUS to perform the procedures. Thus, the presence of centers of excellence and distance, as a geographical factor, can directly impact the use of health services for bariatric surgeries, as indicated by studies conducted in the USA, from 2002 to 2012 and 2003 to 2010, and in Canada, from 2008 to 2015^12^.

Over time and in regional terms, the behavior of socioeconomic, demographic and health management factors explained the index of bariatric surgeries performed by the SUS in Brazil. Descriptive evidence also pointed out that, in 2008 and 2018, the South and Southeast regions of the country concentrated most of the surgical procedures and in the North they were scarce. Therefore, it was important to evaluate, based on evidence, the effects of associated factors, and to understand in which areas the strategic interventions of prevention and control of obesity, especially grade III — severe — are being performed and how these surgeries are effectively distributed.

This study presented the importance of valuing, capacitation and training health professionals who actively participate in bariatric surgeries, and the relevance of expanding the accreditation of hospitals — with acceptable physical, technological, human resources, equipment and financial capacity – so that they can be performed at the state and municipal level, especially in federal units where the offer of the procedure is still small or none.

The results showed that preventive actions of primary care have been effective in reducing the need for the surgical procedure in Brazilian federative units, and it is important to expand the coverage of teams throughout the country. Moreover, characteristics of the population, such as income, schooling and life expectancy, influenced the demand and performance of bariatric surgeries by the public health system. The data obtained by this study can be used for future strategic planning in the management of surgical treatment of obesity in Brazil.

## References

[1] Zubiaurre PR, Bahia LR, Rosa MQM, Assumpção RP, Padoin AV, Sussembach SP (2017). Estimated costs of clinical and surgical treatment of severe obesity in the Brazilian Public Health System. Obes Surg.

[2] Angrisani L, Santonicola A, Iovino P, Ramos A, Shikora S, Kow L (2021). Bariatric Surgery Survey 2018: similarities and disparities among the 5 IFSO chapters. Obes Surg.

[3] Cazzo E, Ramos AC, Chaim EA (2019). Bariatric surgery offer in Brazil: a macroeconomic analysis of the health system’s inequalities. Obes Surg.

[4] Malta DC, Silva AGD, Tonaco LAB, Freitas MIF, Velasquez-Melendez G (2019). Tendência temporal da prevalência de obesidade mórbida na população adulta brasileira entre os anos de 2006 e 2017. Cad Saude Publica.

[5] Ministério da Saúde (BR) (2000). Portaria Nº 196, de 29 de fevereiro de 2000.

[6] Ministério da Saúde (BR) (2007). Portaria nº 492, de 31 de agosto de 2007.

[7] Ministério da Saúde (BR) (2013). Portaria Nº 425, de 19 de março de 2013. Estabelece regulamento técnico, normas e critérios para a Assistência de Alta Complexidade ao Indivíduo com Obesidade.

[8] Ministério da Saúde (BR) (2017). Portaria Nº 5, de 31 de janeiro de 2017. Torna pública a decisão de incorporar o procedimento de cirurgia bariátrica por videolaparoscopia no âmbito do Sistema Único de Saúde - SUS.

[9] Ministério da Saúde (BR), DATASUS (2020). Sistema de Informações Hospitalares do SUS (SIH/SUS).

[10] Xavier DB, Ramalho WM, Silva EN (2017). Spending on bariatric surgery in the Unified Health System from 2010 to 2014: a study based on the specialist hospitals authorized by the Ministry of Health. Obes Surg.

[11] Tonatto AJ, Gallotti FM, Chedid MF, Grezzana TJM, Garcia A (2019). Cirurgia bariátrica no sistema público de saúde brasileiro: o bom, o mau e o feio, ou um longo caminho a percorrer. Sinal amarelo!. ABCD Arq Bras Cir Dig.

[12] Johnson EE, Simpson AN, Harvey JB, Simpson KN (2016). Bariatric surgery implementation trends in the USA from 2002 to 2012. Implement Sci.

[13] Hennings DL, O’Malley TJ, Baimas-George M, Al-Qurayshi Z, Kandil E, DuCoin C (2017). Buckle of the bariatric surgery belt: an analysis of regional disparities in bariatric surgery. Surg Obes Relat Dis.

[14] Doumouras AG, Saleh F, Sharma AM, Anvari S, Gmora S, Anvari M (2017). Geographic and socioeconomic factors affecting delivery of bariatric surgery across high- and low-utilization healthcare systems. Br J Surg.

[15] Bhanderi S, Alam M, Matthews JH, Rudge G, Noble H, Mahon D (2017). Influence of social deprivation on provision of bariatric surgery: 10-year comparative ecological study between two UK specialist centres. BMJ Open.

[16] Billmeier SE, Atkinson RB, Adrales GL (2020). Surgeon presence and utilization of bariatric surgery in the United States. Surg Endosc.

[17] Carvalho ADS, Rosa RDS (2019). Cirurgias bariátricas realizadas pelo Sistema Único de Saúde no período 2010-2016: estudo descritivo das hospitalizações no Brasil. Epidemiol Serv Saude.

[18] Instituto Brasileiro de Geografia e Estatística (2021). Sistema de Contas Regionais (SCR).

[19] Instituto Brasileiro de Geografia e Estatística (2021). Índice Nacional de Preços ao Consumidor Amplo (IPCA).

[20] Instituto Brasileiro de Geografia e Estatística (2021). Pesquisa Nacional por Amostra de Domicílios (PNAD).

[21] Instituto Brasileiro de Geografia e Estatística (2021). Pesquisa Nacional por Amostra de Domicílios (PNAD) Contínua.

[22] Instituto Brasileiro de Geografia e Estatística (2021). Tábuas Completas de Mortalidade.

[23] Souza SA, Silva AB, Cavalcante UMB, Lima CMBL, Souza TC (2018). Obesidade adulta nas nações: uma análise via modelos de regressão beta. Cad Saude Publica.

[24] Ministério da Saúde (BR) (2021). E-Gestor- Informação e Gestão da Atenção Básica.

[25] Ministério da Saúde (BR), DATASUS (2021). Cadastro Nacional de Estabelecimentos de Saúde (CNES). Recursos humanos a partir de agosto de 2007.

[26] Instituto Brasileiro de Geografia e Estatística (2021). Estimativas da População.

[27] Greene WH (2018). Econometric analysis.

[28] Kumanyika SK (2019). A framework for increasing equity impact in obesity prevention. Am J Public Health.

[29] Gomes DCK, Sichieri R, Verly EV, Boccolini CS, Souza AM, Cunha DB (2019). Trends in obesity prevalence among Brazilian adults from 2002 to 2013 by educational level. BMC Public Health.

[30] Pires MRGM, Gottems LBD, Martins CMF, Guilhem D, Alves ED (2010). Oferta e demanda por média complexidade/SUS: relação com atenção básica. Cienc Saude Colet.

