# Supplementary material for: Factors associated with bariatric surgery rates in federative units in Brazil
Source: Rev Saude Publica. 2022 Nov 18;56:117. doi: 10.11606/s1518-8787.2022056004133 (PMC9749656; doi:10.11606/s1518-8787.2022056004133)
Supplement: Translation

# Translation: Fatores associados às taxas de cirurgias bariátricas nas Unidades Federativas do Brasil

**Authors:** Ivan Augusto Cecilio e Silva, Cassia Kely Favoretto, Leticia Xander Russo

**Affiliations:** I Brasil Universidade Federal do Rio Grande do Sul. Faculdade de Ciências Econômicas. Programa de Pós-Graduação em Economia. Porto Alegre, RS, Brasil; II Brasil Universidade Estadual de Maringá. Centro de Ciências Sociais Aplicadas. Departamento de Economia. Maringá, Paraná, PR, Brasil; III Brasil Universidade Federal da Grande Dourados. Faculdade de Administração, Ciências Contábeis e Economia. Departamento de Economia. Dourados, MS, Brasil

**Keywords:** Cirurgia Bariátrica, Disparidades em Assistência à Saúde, Fatores Sociodemográficos, Fatores Socioeconômicos

## Abstract

**OBJETIVO:**

Analisar os fatores socioeconômicos, demográficos e de gestão em saúde associados às taxas de cirurgias bariátricas realizadas pelo Sistema Único de Saúde (SUS) nas unidades federativas (UF) do Brasil.

**MÉTODOS:**

Descrição e análise da taxa de cirurgias bariátricas (por 100 mil habitantes) realizadas pelo SUS em adultos de 18 a 65 anos, nas 27 unidades federativas do Brasil, entre 2008 e 2018; para isso, utilizou-se a metodologia econométrica de painel de contagem com distribuição binomial negativa (*population-averaged*, efeitos fixos e efeitos aleatórios). Investigou-se, também, os fatores socioeconômicos e demográficos, considerando o produto interno bruto (PIB) real *per capita*, a média de anos de estudo de adultos e a expectativa de vida ao nascer, e os da gestão em saúde, tendo em vista a cobertura da atenção básica, a taxa de cirurgiões de aparelho digestivo e a taxa de hospitais credenciados na assistência de alta complexidade (AAC) ao indivíduo obeso no SUS.

**RESULTADOS:**

Em termos regionais, verificou-se grande disparidade na realização de cirurgias bariátricas públicas no Brasil ao longo do período analisado: os procedimentos estão concentrados nas regiões Sul e Sudeste, sendo escassos na região Norte. Além disso, encontrou-se uma relação positiva entre a taxa de cirurgias bariátricas e a expectativa de vida, a taxa de cirurgiões de aparelho digestivo e a taxa de hospitais credenciados na assistência de alta complexidade; havendo, porém, uma associação negativa em relação ao PIB real *per capita*, a média de anos de estudo de adultos e a cobertura da atenção básica.

**CONCLUSÕES:**

No período analisado, o índice de cirurgias bariátricas foi explicado pelos fatores investigados. Portanto, mostrou-se de fundamental importância a formação de profissionais de saúde especializados, o credenciamento de hospitais conforme o marco legal, as ações preventivas da atenção básica, e os fatores socioeconômicos e demográficos, condicionantes da oferta do tratamento cirúrgico pelo Sistema Único de Saúde. Sendo assim, todos fatores relevantes para a formulação de políticas públicas nessa área.

## INTRODUÇÃO

A cirurgia bariátrica é considerada uma intervenção eficaz e de bom custo-benefício para o tratamento da obesidade mórbida em adultos (IMC ≥ 40 kg/m^2^ – Grau III), especialmente para portadores de comorbidades como diabetes e hipertensão^1^. O Brasil, por exemplo, realiza cerca de 17% do total de cirurgias bariátricas no mundo, sendo o segundo país que mais executa esse procedimento, atrás apenas dos Estados Unidos^2^. Entretanto, cerca de 90% desses procedimentos são feitos apenas pelo setor privado, enquanto 75% da população depende exclusivamente do sistema público de saúde^3^. Nesse contexto, a demanda pelo tratamento cirúrgico é crescente, haja vista a tendência de aumento da obesidade mórbida na população brasileira – mais frequente entre mulheres (1,3% em 2006 e 1,9% em 2017) do que entre homens (0,9% em 2006 e 1,4% em 2017) –, chegando a 1,1% no total (homens e mulheres), em 2006, e 1,7%, em 2017^4^.

Quanto às políticas públicas, a cirurgia bariátrica foi incluída na tabela de procedimentos do Sistema Único de Saúde (SUS) em 1999, e no ano seguinte iniciou-se o credenciamento de hospitais para realizá-la^5^. Em 2007, o Ministério da Saúde autorizava três procedimentos responsáveis por reduzirem mais de 60% do excesso do peso inicial dos pacientes^6^: a gastroplastia vertical com banda, o desvio gástrico com Y de Roux e a derivação biliopancreática, ou *switch* duodenal. Já em 2013, o SUS passou a fazer a gastrectomia vertical em manga, ou *sleeve*^7^, e, a partir de 2017, finalmente incorporou a cirurgia bariátrica por videolaparoscopia^8^.

Entre 2008 e 2019, o número de cirurgias bariátricas realizadas pelo SUS, em adultos de 18 a 65 anos, apresentou tendência crescente, passando de 3.158 para 12.432, ou seja, uma expansão de 294% em um pouco mais de uma década. Com base nisso, a maioria das regiões do país apresentou variação absoluta positiva: no Sul, o total expandiu de 1.372 para 8.191 cirurgias (497%), no Centro-Oeste, de 131 para 372 (184%), no Sudeste, de 1.230 para 3.240 (163%), e no Nordeste, de 375 para 587 (57%). Apenas na região Norte o índice de cirurgias foi negativo, diminuindo de 50 para 42, redução de 16% ao longo do período analisado^9^.

Contudo, no SUS, a oferta das cirurgias bariátricas ainda é escassa; o financiamento é limitado e, por isso, muitos hospitais públicos especializados não realizam o número mínimo dessas cirurgias, de 96 por ano ou, em média, 8 por mês, estabelecido pelo Ministério da Saúde^10,11^. Nessa linha, as disparidades no acesso a esse tipo de procedimento no Brasil limitam-no a uma pequena parcela de pacientes que podem se beneficiar do tratamento contra a obesidade mórbida, configurando-se assim em uma questão econômica além de uma questão de saúde.

Pesquisas recentes mostram que há diversos fatores associados ao comportamento das taxas de cirurgias bariátricas no contexto internacional^12^. Ocorre de alguns autores destacarem os condicionantes socioeconômicos e demográficos, como o produto interno Bruto (PIB) *per capita*, a taxa de inflação e a de desemprego, mudanças legislativas, a pobreza e educação^3,12,13^. Outros ressaltam os aspectos de saúde: prevalência de obesidade, Índice de Massa Corporal (IMC), gastos *per capita* em saúde, presença de cirurgiões bariátricos e de hospitais especializados^3,14^.

No Brasil, a literatura atual baseia-se principalmente em análises descritivas da evolução na quantidade e tipos de procedimentos, focando no perfil dos pacientes, isto é, em sua idade, gênero, raça e comorbidades^10,11,17^. Um estudo recente^3^ chegou a analisar a correlação entre a proporção de cirurgias bariátricas realizadas pelo SUS e variáveis macroeconômicas, contudo, o método empregado – coeficiente de correlação de Spearman – permitiu apenas avaliar a intensidade e direção da associação entre duas variáveis. Por isso, o presente estudo busca contribuir para a literatura nacional e internacional ao preencher a lacuna exposta, objetivando analisar os fatores socioeconômicos, demográficos e de gestão em saúde, associados às taxas de cirurgias bariátricas realizadas pelo Sistema Único de Saúde, nas unidades federativas do Brasil entre 2008 e 2018.

## MÉTODOS

Esta pesquisa é descritiva e analítica, utilizando dados do número de cirurgias bariátricas realizadas pelo SUS, em adultos de 18 a 65 anos, nas 27 unidades federativas do Brasil, entre 2008 e 2018. Todos os dados foram coletados de fontes secundárias e sem identificar os indivíduos, conforme os critérios éticos de pesquisa. O Quadro apresenta a descrição das variáveis utilizadas, destacando-se dois grupos de fatores explicativos: socioeconômicos e demográficos (i), e gestão em saúde (ii).

**Quadro:** Descrição das variáveis (dependente e explicativas) usadas na pesquisa, Unidades Federativas do Brasil, 2008–2018.

Variável	Descrição	Fonte
bariatricas	$\text{ Taxa de cirurgias bariátricas realizadas pelo SUS (por }100\text{ mil habitantes) }=\left[\left(\frac{\text{ número de cirurgias bariátricas }}{\text{ (população estimada por UF) }}\right)\times 100.000\right]$	SIH/ Datasus^9^
Fatores socioeconômicos e demográficos		
pibpc	$\text{ PIB real per capita - em mil reais (deflacionado pelo IPCA a preços de 2018) = }\left[\frac{\text{IPCA\_2018}}{\text{IPCA\_ano}}\times \frac{\text{(PIB estadual}÷1.000)}{\text{população estimada por UF}}\right]$	Contas Regionais/ IBGE^18^
anos_estudo	$\text{ Média de anos de estudo em adultos ( }18\text{ a }65\text{ anos) }=\left[\frac{\text{ total de anos de estudo }}{\text{ total de adultos }}\right]$
expec_vida	Esperança de vida ao nascer (em anos).	TCM/IBGE^22^
Fatores de gestão em saúde		
atencao_basica	Percentual da população coberta por equipes de Estratégia Saúde da Família e de atenção básica tradicional, equivalentes e parametrizadas em relação à estimativa populacional (razão decimal).	e-Gestor AB^24^
cirurgioes	$\text{Taxa de cirurgiões do aparelho digestivo que atendem pelo SUS (por 100 mil habitantes) =}\left[\left(\frac{\text{ cirurgiões do aparelho digestivo que atendem pelo SUS }}{\text{ população estimada por UF }}\right)\times 100.000\right]$	CNES/ Datasus^25^
hospitais	$\text{Taxa de hospitais credenciados na assistência de alta complexidade (SUS) ao indivíduo obeso (por 100 mil habitantes) =}\left[\left(\frac{\text{ hospitais credenciados pelo SUS que realizam bariátrica }}{\text{ população estimada por UF }}\right)\times 100.000\right]$	SIH/ Datasus^9^

As informações referente ao número de cirurgias bariátricas foram obtidas por meio do Sistema de Informações Hospitalares^9^ (SIH), disponível no Datasus. Na coleta dos dados, utilizou-se o *software* TabWin-SUS, aplicando-se os seguintes parâmetros: i) unidades federativas de internação por ano de processamento e frequência; ii) período entre janeiro de 2008 e dezembro de 2018; iii) idade entre 18 e 65 anos; iv) procedimentos realizados de gastrectomia com ou sem desvio duodenal (0407010122), de gastroplastia com derivação intestinal (0407010173), de gastroplastia vertical com banda (0407010181), de gastrectomia vertical em manga ou *sleeve* (0407010360), de cirurgia bariátrica por videolaparoscopia (0407010386), e diagnóstico CID10 (categoria) referente a E66 (obesidade). As taxas de cirurgias bariátricas foram obtidas dividindo-se a quantidade realizada de procedimentos, por unidade federativa e ano, pela sua população residente estimada. Essa taxa foi mensurada por 100 mil habitantes, conforme estudos anteriores na área^11,12^.

Cabe ressaltar que a idade indicada no marco legal brasileiro para realizar a cirurgia bariátrica está majoritariamente entre 18 e 65 anos^6^, com algumas exceções não consideradas nesta análise: i) adolescentes entre 16 e 18, se já estiverem consolidadas as epífises de crescimento, e ii) idosos acima de 65 anos, considerando criteriosamente a relação de risco-benefício em cada caso^7^. Por sua vez, os procedimentos supracitados também foram selecionados com base nesse marco, considerando que a gastrectomia vertical em manga foi incluída na tabela de procedimentos do SUS a partir de 2013^7^, e a videolaparoscopia, em 2017^8^.

O PIB real *per capita* (em mil reais) foi usado para captar o nível econômico das unidades federativas brasileiras. Esta variável é importante, pois os ciclos macroeconômicos e o nível da renda podem influenciar no funcionamento do Sistema Único de Saúde e na realização de cirurgias bariátricas^3^. Esse fator foi obtido do Sistema de Contas Regionais^18^ do Instituto Brasileiro de Geografia e Estatística (IBGE) e deflacionado a preços de 2018 (final do período) com base no Índice Nacional de Preços ao Consumidor Amplo (IPCA)^19^.

A média de anos de estudo de adultos entre 18 e 65 anos representou o fator social, no qual se mostra a desigualdade educacional nas unidades federativas do Brasil. Até mesmo porque o nível educacional pode influenciar os índices de obesidade mórbida da população e a demanda por tratamentos como as cirurgias bariátricas^12^. No que se refere à fonte dos dados dessa variável, utilizou-se, para os anos de 2008, 2009, e de 2011 a 2015, a Pesquisa Nacional por Amostra de Domicílios (PNAD)^20^, e, para 2016, 2017 e 2018, a PNAD Contínua Anual^21^. Para 2010, devido à indisponibilidade dos dados, adotou-se como estimativa a média entre o ano imediatamente anterior e o seguinte.

Já o fator demográfico correspondeu à expectativa de vida ao nascer, em anos, a qual tem como base as tábuas completas de mortalidade, por ano e unidade da federação, conforme informações do IBGE^22^. A expectativa de vida é fundamental para explicar a proporção de adultos obesos e, portanto, a realização de cirurgias bariátricas, na medida em que os cuidados com a saúde e bem-estar impactam também no processo de envelhecimento populacional^23^.

A cobertura da atenção básica é um fator de gestão em saúde obtido pelo Sistema de Informação e Gestão da atenção básica^24^ (e-Gestor), representando o percentual da população que é atendida por equipes da Estratégia Saúde da Família e da atenção básica tradicional, essenciais para prevenção e diagnóstico precoce da obesidade nas unidades federativas.

A taxa de cirurgiões de aparelho digestivo que atendem pelo SUS, mensurada por 100 mil habitantes, é uma forma de analisar a capacidade do sistema público em ofertar cirurgias bariátricas, condicionadas à disponibilidade de especialistas no procedimento, seguindo a literatura^16^. Esses dados foram obtidos do Cadastro Nacional de Estabelecimentos de Saúde^25^ (CNES) para recursos humanos, que apresentou uma grande variabilidade mensal na taxa citada, sendo necessário o uso do cálculo da sua média anual.

A taxa de hospitais credenciados à assistência de alta complexidade (AAC) ao indivíduo obeso (por 100 mil habitantes) representa a infraestrutura – recursos humanos e equipamentos – necessária para realização de procedimentos de cirurgia bariátrica, conforme Portaria nº 425 de 2013^7^ e a literatura internacional^12^. Os dados foram coletados do SIH^9^ por meio do *software* TabWin-SUS, aplicando o parâmetro de frequência segundo hospital.

Finalmente, a taxa populacional residente por unidade federativa e ano de análise, conforme estimativas do IBGE^26^, foi utilizada para calcular os índices de cirurgias bariátricas, de cirurgiões de aparelho digestivo que atendem pelo SUS e de hospitais credenciados à assistência de alta complexidade ao indivíduo com obesidade. Isso foi realizado dividindo essas variáveis pelo indicador de intensidade de cada uma das unidades, ou seja, pelo número de habitantes.

Por hipótese, espera-se que os fatores socioeconômicos, demográficos e de gestão em saúde estejam relacionados com o índice de cirurgias bariátricas realizadas pelo SUS entre 2008 e 2018; além disso, que expliquem as diferenças regionais observadas em cada unidade federativa, considerando a quantidade dessas cirurgias.

Devido às características do fenômeno estudado, como a presença de heterogeneidade entre as unidades da federação brasileira ao longo do tempo (dados longitudinais), a forte concentração de “zeros” – unidades que não realizaram nenhuma cirurgia – e a super-dispersão – variância maior que a média –, optou-se pela metodologia econométrica de painel de contagem com distribuição binomial negativa (*population-averaged*, efeitos fixos e efeitos aleatórios)^27^. Sendo assim, o intuito deste estudo é modelar a quantidade contável de cirurgias bariátricas realizadas por 100 mil habitantes, considerando a limitação imposta pela escassez de outras metodologias para o painel de contagem inflacionado de “zero”, mais comumente aplicadas apenas na forma *cross-section* – para um único ano. Por fim, todos os dados da pesquisa foram organizados em planilha eletrônica e as estimações foram realizadas a partir do *Software* Stata 13.

## RESULTADOS

Na Tabela 1 está apresentada a estatística descritiva das variáveis – dependente e explicativas – para as unidades federativas do Brasil, no período de 2008 a 2018. A taxa média de cirurgias bariátricas feitas pelo SUS foi de 2,44 por 100 mil habitantes, com valor máximo de 58,46 por 100 mil, registrado no Paraná em 2018. O valor mínimo 0, ou seja, nenhuma cirurgia, ocorreu em 85 observações durante o período, sendo 10 unidades da federação, em 2008, e 7, em 2018. Nota-se que o desvio padrão é maior que a média (6,328 > 2,439). Portanto, a variância – desvio padrão ao quadrado – também o é, o que indica super-dispersão dos dados. Assim, a dispersão relativa foi alta – o coeficiente de variação foi igual a 259,45% –, indicando que existem desigualdades na realização desse procedimento entre as áreas analisadas.

**Tabela 1:** Estatística descritiva das variáveis (dependente e explicativas) usadas na pesquisa, Unidades Federativas do Brasil, 2008–2018 (n = 297).

Variável dependente/fatores	Média	Desvio padrão	Valor mínimo	Valor máximo	CV (%)
Taxa de cirurgias bariátricas no SUS (por 100 mil habitantes)	2,439	6,328	0,000	58,455	259,45
Fatores socioeconômicos e demográficos					
PIB real *per capita* (em mil reais)	28,082	15,304	9,160	89,780	54,50
Escolaridade média de adultos (em anos)	9,158	0,936	6,900	11,700	10,22
Expectativa de vida ao nascer (em anos)	73,539	2,626	68,100	79,700	3,57
Fatores de gestão em saúde					
Cobertura da atenção básica (razão decimal)	0,763	0,130	0,405	0,998	17,04
Taxa de cirurgiões do aparelho digestivo no SUS (por 100 mil habitantes)	0,776	0,622	0,067	2,679	80,15
Taxa de hospitais credenciados na AAC para obesos no SUS (por 100 mil habitantes)	0,035	0,037	0,000	0,159	105,71

CV: coeficiente de variação; AAC: assistência de alta complexidade.

Com relação aos fatores associados, verificou-se que o PIB real *per capita* médio foi de R$ 28.082,00 entre 2008 e 2018, com valores mínimo de R$ 9.160,00, no Piauí em 2008, e máximo de R$ 89.780,00, no Distrito Federal em 2010. A escolaridade média em adultos, no período analisado, foi de aproximadamente 9,2 anos de estudo, sendo o menor valor desse fator (6,9 anos) observado em Alagoas em 2008, e o maior (11,7 anos), no Distrito Federal em 2018. Já a expectativa de vida média – fator demográfico – correspondeu a 73,5 anos, atingindo a idade mínima de 68,1 anos no Maranhão em 2008, e máxima de 79,7 anos no estado de Santa Catarina em 2018. O coeficiente de variação desses três fatores foi 54,50%, 10,22% e 3,57%, respectivamente, apontando que a principal disparidade entre as unidades federativas foi econômica.

Com relação aos fatores de gestão em saúde, entre 2008 e 2018, observou-se que a cobertura da atenção básica média foi de 76,3%, sendo a mínima de 40,6% no Distrito Federal, em 2008, e máxima de 99,8% no Piauí, em 2018. A taxa média de cirurgiões de aparelho digestivo no SUS foi de 0,77 por 100 mil habitantes entre 2008 e 2018, com valor mínimo de 0,067 por 100 mil habitantes em Rondônia, no ano de 2008, e máximo de 2,67 por 100 mil habitantes no Paraná, em 2018. Por sua vez, o fator taxa de hospitais credenciados na assistência de alta complexidade para obesos no SUS registrou média de 0,035 por 100 mil habitantes, com o valor mais alto dessa variável de 0,159 por 100 mil habitantes, identificado no Paraná em 2018. Desse modo, os resultados dos coeficientes de variação do primeiro (17,04%), segundo (80,15%) e terceiro (105,71%) condicionantes mostraram que o setor da saúde apresentou diferenças de comportamento entre as unidades da federação brasileira.

Em termos regionais, foi vista uma desigualdade na taxa de cirurgias bariátricas ao longo de todo o período. A Figura 1 apresenta a distribuição das taxas no mapa do Brasil para os anos de 2008 e 2018, mostrando a evolução geográfica do início ao final da análise. A concentração é maior nas regiões Sul e Sudeste, principalmente em 2018, enquanto a região Norte possui menor oferta do procedimento. Destaca-se que os estados do Acre, Rio Grande do Norte, Paraíba, Sergipe e Goiás não realizavam cirurgia bariátrica pelo SUS em 2008; contudo, em 2018, já a tinham implementado. Pará e Mato Grosso executaram esse tipo de procedimento em 2008, mas deixaram de fazê-lo no último ano de análise. Já outros cinco estados, Rondônia, Amazonas, Roraima, Amapá e Piauí, não registraram nenhuma cirurgia bariátrica pública durante os anos estudados.

**Figura 1:** Mapas *quantile* da taxa de cirurgias bariátricas (por 100 mil habitantes), Unidades Federativas do Brasil, 2008 e 2018.

Na Figura 2, apresenta-se os diagramas de dispersão entre a variável dependente e cada um dos fatores explicativos. Nela se observa que o índice de cirurgias bariátricas realizadas pelo SUS apresentou um ajuste linear positivo com os fatores socioeconômicos, demográficos e de gestão em saúde, exceto com a cobertura da atenção básica.

**Figura 2:** Diagramas de dispersão entre a variável dependente e as explicativas, Unidades Federativas do Brasil, 2008–2018 (n = 297). A variável “bariatricas” refere-se a taxa de cirurgias bariátricas realizadas pelo SUS (por 100 mil habitantes), “anos_estudo” é a média de anos de estudo em adultos (em anos); “expec_vida” é a expectativa de vida (em anos); “atencao_basica” é a cobertura populacional da atenção básica (em razão decimal); “cirurgioes” é a taxa de cirurgiões do aparelho digestivo no SUS (por 100 mil habitantes); “hospitais” é a taxa de hospitais credenciados na AAC para obesos no SUS (por 100 mil habitantes).

Na Tabela 2 estão apresentados os resultados para painel de contagem com distribuição Binomial Negativa e com erro padrão robusto (modelos *population-averaged*, efeitos aleatórios e efeitos fixos). No painel de contagem por efeitos fixos, os cinco estados, Rondônia, Amazonas, Roraima, Amapá e Piauí, não entraram na estimativa por causa da variável dependente ser composta apenas de zeros em todas as 55 observações; logo, o número total de observações foi menor neste modelo (n = 242).

**Tabela 2:** Resultados das estimações dos modelos em painel de binomial negativa com erro padrão robusto, Unidades Federativas do Brasil, 2008–2018.

Variáveis	Population-averaged	Efeitos aleatórios	Efeitos fixos
Constante	-26,587^c^	-13,035	13,437
	(7,511)	(373,103)	(379,157)
PIB *per capita* (real)	-0,052^c^	-0,045^c^	-0,042^b^
	(0,012)	(0,011)	(0,021)
Anos de estudo em adultos (média)	-0,180	-0,362^a^	-0,102
	(0,232)	(0,219)	(0,344)
Expectativa de vida	0,429^c^	0,487^c^	0,092
	(0,125)	(0,089)	(0,324)
Cobertura da atenção básica	-3,933^c^	-2,774^b^	-3,676
	(1,254)	(1,290)	(2,403)
Taxa de cirurgiões do aparelho digestivo no SUS	0,674^c^	0,642^c^	0,671^b^
	(0,218)	(0,204)	(0,310)
Taxa de hospitais credenciados na AAC para obesos no SUS	16,252^c^	13,483^c^	10,518^c^
	(3,253)	(2,339)	(2,486)
*Dummies* por ano	Sim	Sim	Sim
Erro padrão	Robusto	Robusto	Robusto
n	297	297	242
Teste de Hausman	-	0,978	

SUS: Sistema Único de Saúde; AAC: assistência de alta complexidade.

Erro padrão em parênteses:

^a^ p < 0,1

^b^ p < 0,05

^c^ p < 0,01

O teste de Hausman não foi significativo (p > 0,05), portanto, o modelo de efeitos aleatórios foi mais adequado ao de efeitos fixos para análise dos resultados. No primeiro modelo, todos os fatores foram estatisticamente significativos no nível de 10%. O PIB *per capita*, os anos de estudo e a cobertura da atenção básica tiveram associações negativas com o índice de cirurgias bariátricas. Todavia, a expectativa de vida, a taxa de cirurgiões de aparelho digestivo e a taxa de hospitais credenciados na Assistência de Alta Complexidade tiveram associações positivas.

## DISCUSSÃO

A partir dos resultados da seção anterior, constatou-se o efeito negativo do fator econômico – PIB real *per capita* – em relação ao índice de cirurgias bariátricas por unidades federativas. Isso significa um aumento no nível econômico associado à redução na oferta desse procedimento, possivelmente porque as ações estratégicas de prevenção à obesidade tendem a ser adotadas nas áreas mais economicamente desenvolvidas, em detrimento das populações de baixa renda^28^ – mesmo assim, um outro estudo^3^ não encontrou relevante correlação entre a proporção de cirurgias bariátricas públicas e a taxa de variação anual do PIB no Brasil entre 2003 e 2017. Entretanto, o presente artigo difere da abordagem anterior em alguns aspectos importantes: na utilização do painel de contagem; na incorporação de diversas variáveis, inclusive *dummies* para controle de cada ano; e na realização da análise a nível das unidades federativas, captando as particularidades locais.

Por um lado, a escolaridade, logo, média de anos de estudo, também se associou negativamente à oferta de cirurgias bariátricas pelo SUS em cada unidade federativa, o contrário do que aponta um estudo^12^ realizado para os EUA, de 2002 a 2012, no qual se demonstrou que o fator educacional não foi significativo para o processo de difusão de cirurgias bariátricas nos estados americanos. Sabe-se que níveis mais baixos de escolaridade podem ser fator de risco para a obesidade, principalmente para as mulheres^29^; esse público é o que mais procura pelos procedimentos ofertados pelo SUS, compreendendo 85% dos casos^17^.

Por outro, o fator demográfico, tendo em conta a expectativa de vida, exerceu impacto positivo e significativo sobre o índice de cirurgias bariátricas nas unidades federativas brasileiras, entre 2008 e 2018. Conforme a população envelheceu, medidas de cuidados com a saúde e bem-estar tornaram-se necessárias, principalmente na questão da obesidade mórbida grave e da oferta de cirurgias^23^. Esse efeito implica que, se o processo de envelhecimento da população nas unidades da federação, ao longo do tempo, não é saudável – consequência do sedentarismo e da má alimentação ao decorrer da vida –, a incidência de obesidade mórbida tende a aumentar, expandindo a demanda pelo procedimento médico.

Nessas circunstâncias, o aumento da cobertura de equipes da atenção básica contribuiu para a redução das taxas de cirurgias bariátricas nas unidades federativas, reforçando a importância dessa estratégia de política pública para a prevenção e controle dos índices de obesidade mórbida no país. A eficácia da abordagem integral e intrasetorial sobre a obesidade no SUS, no âmbito das linhas de cuidado para doenças crônicas, reforça o vínculo entre a cobertura da atenção básica e a demanda por serviços de média ou alta complexidade^30^.

O impacto positivo da taxa de cirurgiões de aparelho digestivo sobre o índice de cirurgias bariátricas realizadas pelo Sistema Único de Saúde nas unidades federativas indicou que esse fator é de fundamental importância para a oferta do procedimento no país, em especial no contexto regional. Logo, a contratação, valorização e treinamento desses profissionais no sistema público de saúde tornam-se essenciais para minimizar o problema de obesidade mórbida^11^. Esses resultados estão em conformidade com o estudo sobre o caso dos EUA no ano de 2013, em que o tratamento eficaz para obesidade mórbida pareceu ser limitado pela quantidade de cirurgiões capacitados entre as diferentes áreas desse país^16^.

Igualmente, a taxa de credenciamento de hospitais na assistência de alta complexidade para indivíduos obesos mostrou associação positiva com os índices de cirurgias bariátricas realizadas no sistema público de saúde do Brasil. Isso indica que a expansão dos hospitais especializados e credenciados nos estados é fator essencial para ampliar a oferta do procedimento, particularmente nas regiões menos assistidas^10^. Nem mesmo aqueles estados onde não se registrou nenhuma cirurgia bariátrica pública ao longo do período analisado – Rondônia, Amazonas, Roraima, Amapá e Piauí – estão isentos de indivíduos portadores de obesidade mórbida, apesar de não apresentarem serviços especializados e credenciados pelo SUS para realização dos procedimentos. Sobre isso, a presença de centros de excelência e a distância, como fator geográfico, podem impactar diretamente no uso dos serviços de saúde para a realização de cirurgias bariátricas, como apontaram estudos realizados nos EUA, de 2002 a 2012 e 2003 a 2010, e no Canadá, de 2008 a 2015^12^.

Isto posto, ao longo do tempo e em termos regionais, observou-se que o índice de cirurgias bariátricas realizadas pelo SUS no Brasil foi explicado pelo comportamento de fatores socioeconômicos, demográficos e de gestão em saúde. As evidências descritivas também apontaram que, nos anos de 2008 e 2018, houve concentração dos procedimentos cirúrgicos nas regiões Sul e Sudeste do país e escassez na região Norte. Assim sendo, revelou-se importante avaliar, com base em evidências, os efeitos de fatores associados, além de compreender em quais áreas as intervenções estratégicas de prevenção e controle da obesidade, principalmente de grau III – grave –, estão sendo realizadas e como essas cirurgias estão efetivamente distribuídas.

Reitera-se que, a partir deste estudo, é possível inferir a importância da valorização, capacitação e treinamento dos profissionais de saúde que participam ativamente das cirurgias bariátricas, e deduzir a relevância da expansão do credenciamento de hospitais – com capacidade física, tecnológica, de recursos humanos, de equipamentos e financeira aceitáveis – para que elas sejam realizadas em nível estadual e municipal, sobretudo nas unidades federativas onde a oferta do procedimento ainda é pequena ou nula.

Em suma, os resultados demonstram que as ações preventivas da atenção básica têm sido eficazes para reduzir a necessidade do procedimento cirúrgico nas unidades federativas brasileiras, sendo importante expandir a cobertura de equipes pelo país. Além disso, características da população, tais como a renda, a escolaridade e a expectativa de vida, mostraram-se diretamente influentes na demanda e realização de cirurgias bariátricas pelo sistema público de saúde. Posto isto, os dados obtidos por meio deste estudo podem ser utilizados para futuro planejamento estratégico na gestão do tratamento cirúrgico da obesidade no Brasil.
